# A State Veterinarian for California

**Published:** 1895-01

**Authors:** 


### A STATE VETERINARIAN FOR CALIFORNIA.

CALIFORNIA is about to consider the wisdom of establishing the office of State Veterinarian with Assistant Veterinarians in the counties of that State. An act has been drawn as follows:

Section 1 provides for the appointment by the Governor, with the approval of the State Board of Health, of a State Veterinarian who shall be a graduate and shall hold office for a term of four years.

Sec. 2 provides for said Veterinarian taking the usual oath of office as a State officer.

Sec. 3 provides for the salary and necessary expenses of the office.

Sec. 4 provides that it shall be the duty of the Board of Supervisors, with the consent and approval of the State Veterinarian, to appoint competent and skilled County Veterinarians, and that said Veterinarians shall hold a license of the State Veterinary Medical Board to hold office for four years, to be removed for good cause by order of the State Veterinarian or the Board of Supervisors.

Sec. 5 provides for County Veterinarians taking the necessary oath of office.

Sec. 6 provides for salary and expenses.

Sec. 7 provides for the quarantining of all animals by and with the consent of the State Board of Health and that the duties of County Veterinarians shall be prescribed by the State Veterinarians.

Sec. 8 provides for all persons having diseased animals supposed to be dangerous, contagious, or infectious to report the same immediately to the County Veterinarian, and through him to the State Veterinarian.

Sec. 9 refers to the County Veterinarian keeping a record of all cases so reported and to take such measures as may be necessary until relieved by the State Veterinarian.

Sec. 10 provides for the State Veterinarian taking control of all cases of infectious or contagious diseases among the domestic animals, and to adopt such measures for their suppression and eradication as may be required, as well as the quarantining of places and districts, and all other regulations as to handling, treating, feeding, etc., that may be necessary. Also the slaughtering and appraisement of those necessary to control the diseases and protect the health of the public and domestic animals in such localities.

Sec. 11 provides for reporting the same to the Governor and the issuing of any proclamation that may be necessary.

Sec. 12 provides for the appraisement of animals destroyed, first by the State Veterinarian, and, if this is not satisfactory, by three appraisers. The appraisement for horses and for mules shall not in any case exceed seventy-five dollars ($75). Twenty dollars ($20) for cattle.

Sec. 13 provides for the method of reimbursing the person or persons whose animals are destroyed, and states that they shall not be paid more than two-thirds or less than one-half of the appraised value.

Sec. 14 provides for the contingency of animals being brought into the State in a diseased condition for the purpose of being slaughtered, and securing reimbursement for the same.

Sec. 15 provides for any violation of the act in the shipping, selling, etc., of any animal affected with any contagious or infectious diseases when known or when said animals have been placed in quarantine, with a fine and penalty for the same.

Sec. 16 is evidently aimed at railroad corporations or others who may knowingly bring into the State diseased animals, and a fine and penalty to be imposed.

Sec. 17 provides a penalty for interfering with the duties of State or County Veterinarians.

Sec. 18 provides a penalty for the violation of any of the provisions of this act.

Sec. 19 provides for expenses for supplies and materials necessary in the work to be done.

Sec. 20 provides that all fines recovered in the provisions of the act shall be paid into the county treasury of the county where the same are collected.

Sec. 21 provides for the State or County Veterinarian having the power to call upon sheriffs, deputies, or other officers to aid in enforcing the provisions of the act.

Sec. 22, which is something entirely new, provides for the work as State work, the right to co-operate with any board or commission of the United States that may be provided by any present or future act of Congress for dealing with contagious and infectious diseases among domestic animals.

Secs. 23, 24, 25 provide for the annual report of the State Veterinarian, for the act to go into effect, and to repeal all other acts inconsistent with the one proposed.

This is followed by a summary of the total number of horses, mules, milch cows, oxen, sheep, and swine in California, which is in round numbers 6,200,000, and the total value, $54,000,000. It is estimated that the annual loss in California from contagious and infectious diseases is $1,600,000. Reference is then made to the various States having laws for the control of all infectious diseases among domestic animals, in which twenty-six States are enumerated, closing with an appeal for support of the act.

